# Validation of anterior ankle soft tissue dynamics and shear modulus for anterior ankle impingement syndrome after ankle fracture surgery

**DOI:** 10.1038/s41598-024-56671-5

**Published:** 2024-03-11

**Authors:** Haruki Osanami, Hiroshi Akuzawa, Kodai Sakamoto, Hirotake Yokota, Ryo Hirabayashi, Chie Sekine, Tomonobu Ishigaki, Mutsuaki Edama

**Affiliations:** 1 Institute for Human Movement and Medical Sciences, Niigata University of Health and Welfare, Shimami-cho, 1398, Kita-ku, Niigata City, Niigata 950-3198 Japan; 2 Department of Rehabilitation, Keiyu Orthopaedic Hospital, 2267 Akoda, Tatebayashi, Gunma 374-0013 Japan

**Keywords:** Anterior ankle soft tissue, Anterior ankle impingement syndrome, Ankle fracture, Ultrasonography, Shear-wave elastography, Anatomy, Medical research, Risk factors

## Abstract

Anterior ankle impingement syndrome (AAIS) has been reported to account for a high percentage of complications following ankle fracture surgery. The soft tissue etiology of AAIS is thought to be thickening and inflammation of the anterior ankle soft tissues intervening anteriorly at the tibiotalar joint, causing pain and functional limitation during dorsiflexion. However, the effects of anterior ankle soft tissue dynamics and stiffness on AAIS have yet to be clarified. This study aimed to determine the relationship between AAIS and the anterior ankle soft tissue thickness change ratio and shear modulus using ultrasonography (US). The participants were 20 patients with ankle joint fractures (AO classification A, B) who had undergone open reduction and internal fixation and 20 healthy adults. The evaluation periods were 3 months and 6 months postoperatively. US was used to delineate the tibialis anterior tendon, extensor hallucis longus tendon, and the extensor digitorum longus tendon over the talus and tibia on a long-axis image. Anterior ankle soft tissue thickness was measured as the shortest distance from the most convex part of the talus to the tendon directly above it. The Anterior ankle soft tissue thickness change ratio was determined by dividing the value at 0° dorsiflexion by the value at 10° plantarflexion. The same images as for the anterior soft tissue thickness measurement were drawn for the shear modulus measurement, and the average shear modulus (kPa) was calculated using shear-wave elastography. There was no significant difference in the thickness change ratio between the postoperative and healthy groups. Compared with the healthy group, the shear modulus was significantly higher at 3 and 6 months in the postoperative group (p < 0.01). The shear elastic modulus at 6-month postoperative group was significantly lower than at 3-month postoperative group (p < 0.01). Anterior ankle joint soft tissue stiffness may increase after surgery for an ankle fracture.

## Introduction

Ankle fractures are common traumatic injuries, with a reported incidence of 169 per 100,000^1,2^. A systematic review of long-term outcomes after ankle fracture surgery reported that 20.7% of patients did not have a good outcome, even with good repair^3^. In addition, 30–40% of postoperative ankle fracture patients experience ongoing pain for more than 3 months after surgery^4^. In a report investigating complications after ankle fracture surgery, anterior ankle impingement syndrome (AAIS) was found in 45% of cases, the highest rate^5^. However, evaluation and treatment of AAIS after ankle fracture surgery have not been adequately studied.

AAIS, defined as anterior ankle pain and limited range of motion (ROM) during ankle dorsiflexion, is broadly classified into bony and soft tissue restrictions^6^. For bony restrictions, osteophyte severity was not found to be correlated with pain duration or the degree of sports activity^7^. The soft tissue etiology of AAIS is thought to be thickening and inflammation of the anterior ankle soft tissues intervening anteriorly at the tibiotalar joint, causing pain and functional limitation during dorsiflexion^8^. Therefore, evaluation and treatment of patients with AAIS should focus on anterior ankle soft tissues.

Anatomical studies of anterior ankle soft tissue anatomy have reported that the anterior tibiotalar joint is composed of adipose tissue, synovium, and blood vessels^9^. Furthermore, fresh-frozen cadaver reports suggested that anterior ankle soft tissue, including adipose tissue, causes impingement of the anterior tibiotalar joint during ankle dorsiflexion^10^. Therefore, dynamic evaluation of anterior ankle soft tissues during dorsiflexion is important. Magnetic resonance imaging (MRI) is generally used to evaluate anterior ankle soft tissue in AAIS^11,12^. However, ultrasonography (US) has recently made possible the dynamic evaluation of anterior ankle soft tissue during loading. US can detect anterior ankle soft tissue thickening and synovitis, and ultrasound-guided therapy reportedly improves pain and function in AAIS^13^. However, these were mostly case reports; anterior ankle soft tissue stiffness and dynamic evaluation have not been adequately studied^13,14^. In the knee joint, the infrapatellar fat pad (IFP) is associated with the synovium, causing fibrosis and inflammation, which is thought to cause the pain^15,16^. Therefore, several studies of IFP stiffness and dynamic assessment have been reported^17–19^. Kitagawa et al.^17^ evaluated the IFP thickness change ratio in anterior cruciate ligament (ACL)-reconstructed knees at 90° and 10° of knee flexion and defined the thickness change ratio as dynamic. Katayama et al.^18^ measured the IFP’s shear modulus in healthy adults using shear-wave elastography and evaluated IFP stiffness. In addition, Satake et al.^19^ reported that they observed a percentage of maximal fibrosis to increase as the shear wave speed of IFP increased. The results suggest the need to evaluate IFP dynamics and shear modulus. However, while there have been reports investigating anterior ankle soft tissue morphology^20^, there have not been enough reports investigating dynamics and shear modulus. Therefore, we believe that evaluation of the dynamics and shear modulus evaluation of the IFP can be applied to the anterior ankle soft tissue to determine its effect on AAIS.

This study aimed to determine the anterior ankle soft tissue dynamics and shear modulus of AAIS after ankle fracture surgery, examining the following three hypotheses. First, the anterior ankle soft tissue thickness change ratio is lower after ankle fracture surgery compared to healthy controls. The thickness change ratio was assumed to decrease in the postoperative course. The shear modulus of the anterior ankle soft tissue was assumed to be higher after ankle fracture surgery than in a healthy ankle and to decrease during the postoperative course. Second, the anterior ankle soft tissue thickness change ratio is lower, and the shear modulus is higher in patients with AAIS after ankle fracture compared to asymptomatic patients. Third, a lower anterior ankle soft tissue thickness change ratio and higher shear modulus are associated with a smaller dorsiflexion ROM after ankle fracture surgery.

## Results

### Anterior ankle soft tissue thickness change ratio and reliability of shear modulus measurement

ICC (1,3), ICC (2,3), and minimal detectable difference at the 95% confidence interval (MDD95%) for intersession measurements are shown in Table 1. ICC (1,3) for the thickness change ratio and shear modulus ranged from 0.88 to 0.93 (almost perfect). ICC (2,3) for the thickness change ratio and shear modulus ranged from 0.83 to 0.98 (almost perfect).

**Table 1:** Intersession reliability of the anterior ankle soft tissue thickness change ratio, shear modulus measurements, and MDD95%.

	Test 1	Test 2	ICC (1,3)	MDD 95%	Reliability
Thickness change ratio (%)	127.2 ± 13.9	128.6 ± 14.8	0.93	10.8	Almost perfect
Shear modulus					
10° plantarflexion (kPa)	12.1 ± 3.6	12.3 ± 3.3	0.89	3.2	Almost perfect
0° dorsiflexion (kPa)	10.2 ± 1.7	10.3 ± 2.0	0.88	1.8	Almost perfect

	Rater 1	Rater 2	ICC (2, 3)	MDD 95%	Reliability
Thickness change ratio (%)	127.0 ± 13.8	123.8 ± 12.2	0.92	10.5	Almost perfect
Shear modulus					
10° plantarflexion (kPa)	12.1 ± 3.6	12.3 ± 3.8	0.98	1.5	Almost perfect
0° dorsiflexion (kPa)	10.2 ± 1.7	10.1 ± 2.1	0.83	2.2	Almost perfect

*MDD95%* minimal detectable difference at the 95% confidence interval, *ICC* intraclass correlation coefficient.

### Comparison of the thickness change ratio and shear modulus between healthy and postoperative groups

There were no significant differences in demographic data between the healthy and postoperative groups. Compared to the healthy group, there was no significant difference in the thickness change ratio in the 3-month (p = 0.35) and 6-month (p = 0.94) postoperative groups. There was no significant difference in the thickness change ratio between the 3-month and 6-month postoperative groups (p = 0.74). Shear modulus was significantly higher at 3 and 6 months in the postoperative group than in the healthy group (p < 0.01). Shear modulus was significantly lower in the 6-month postoperative group than in the 3-month postoperative group (p < 0.01) (Table 2).

**Table 2:** Comparison of thickness change ratio, shear modulus, and dorsiflexion range of motion between the healthy group and the postoperative group.

	Healthy group	3-Month postoperative group	6-Month postoperative group
Thickness			
10° plantarflexion (mm)	11.1 ± 1.6	12.2 ± 2.2	11.7 ± 1.6
0° dorsiflexion (mm)	13.7 ± 1.8	14.6 ± 1.8	14.1 ± 2.0
Thickness change ratio (%)	124.1 ± 11.2	121.0 ± 9.8^a^	120.9 ± 6.3^b,c^
Shear modulus			
10° plantarflexion (kPa)	14.5 ± 6.5	42.1 ± 22.6^d^	28.2 ± 14.0^e,f^
0° dorsiflexion (kPa)	11.4 ± 2.8	35.2 ± 19.8^ g^	22.8 ± 9.4^ h,i^
Dorsiflexion ROM (°)	18.2 ± 5.1	12.0 ± 7.2^j^	13.7 ± 7.4^ k,l^

Values are presented as means ± SD.

*ROM* range of motion.

^a^*P* = 0.35 vs Healthy group.

^b^*P* = 0.94 vs Healthy group.

^c^*P* = 0.74 vs 3-Month postoperative group.

^d^*P* = 0.001 vs Healthy group.

^e^*P* < 0.001 vs Healthy group.

^f^*P* < 0.001 vs 3-Month postoperative group.

^g^*P* = 0.001 vs Healthy group.

^h^*P* < 0.001 vs Healthy group.

^i^*P* = 0.001 vs 3-Month postoperative group.

^j^*P* = 0.003 vs Healthy group.

^k^*P* = 0.03 vs Healthy group.

^l^*P* = 0.15 vs 3-Month postoperative group.

### Comparison of the thickness change ratio and shear modulus between ankle impingement sign-positive and sign-negative groups

At 3 months postoperatively, 6 were in the positive group and 14 in the negative group. At 6 months postoperatively, there were 3 in the positive group and 17 in the negative group. There were no significant differences in demographic data between the positive and negative groups. Comparison of the positive and negative groups at 3 and 6 months postoperatively showed no significant differences in the thickness change ratio and shear modulus (Table 3).

**Table 3:** Comparisons of the thickness change ratio and shear modulus in the positive and negative groups for ankle impingement signs.

Ankle impingement sign	3-Month postoperative group		P-value between groups	6-Month postoperative group		P-value between groups
	Positive group (n = 6)	Negative group (n = 14)		Positive group (n = 3)	Negative group (n = 17)	
Thickness change ratio (%)	123.9 ± 10.1	119.8 ± 9.8	0.39	119.9 ± 9.5	128.6 ± 6.0	0.77
Shear modulus						
10° plantarflexion (kPa)	35.2 ± 27.3	45.0 ± 20.6	0.39	29.7 ± 13.8	29.6 ± 14.5	0.76
0° dorsiflexion (kPa)	28.5 ± 21.4	38.1 ± 19.2	0.24	23.8 ± 14.9	24.0 ± 9.2	0.83

Values are presented as means ± SD.

### Correlations of ankle dorsiflexion ROM, thickness change ratio, and shear modulus

No significant correlations of ankle dorsiflexion ROM with thickness change ratio and shear modulus were found (Table 4).

**Table 4:** Correlations of dorsiflexion ROM with the thickness change ratio and shear modulus.

	3-Month postoperative group dorsiflexion ROM		6-Month postoperative group dorsiflexion ROM	
	r	P-value	r	P-value
Thickness change ratio (%)	− 0.12	0.61	− 0.11	0.64
Shear modulus				
10° plantarflexion (kPa)	− 0.30	0.20	− 0.26	0.25
0° dorsiflexion (kPa)	− 0.40	0.08	− 0.38	0.10

*r* represents the correlation coefficient, *ROM* range of motion.

## Discussion

In this study, the anterior ankle soft tissue thickness change ratio and shear modulus were investigated in postoperative patients with ankle fractures. To the best of our knowledge, this is the first study to focus on the anterior ankle soft tissue and measure the thickness change ratio and shear modulus.

The first was that there was no significant difference in the ratio of change in thickness between the postoperative group and the healthy group. Second, the postoperative group showed a higher shear modulus than the healthy group, and the shear modulus was significantly higher than that of the healthy group, although a decrease in shear modulus was observed with postoperative course. Third, the rate of change in anterior ankle soft tissue thickness and shear modulus showed no significant difference between the ankle impingement sign positive and negative groups, and no significant correlation with ankle dorsiflexion range of motion.

Regarding the first finding, there was no significant difference in the ratio of change in thickness in the postoperative group compared to the healthy group. Furthermore, there was no significant difference in the thickness change ratio between the 3 months and 6 months postoperative groups. Therefore, we hypothesized that anterior ankle soft tissues show the same dynamics as healthy soft tissues after ankle fracture surgery. A previous study by Shiraishi et al.^21^ compared the IFP thickness change ratio between anterior cruciate ligament-reconstructed and non-reconstructed knees and reported no significant difference in the IFP thickness change ratio. However, Kitagawa et al.^17^. reported a significant difference in the rate of change in IFP thickness at 90° and 0° knee joint flexion. It has been reported that the anterior soft tissues of the ankle joint are compressed anterior of the joint by 15° dorsiflexion of the ankle joint^10^, and that AAIS is also caused by dorsiflexion of the ankle joint during loading^22^. Based on these facts, it is possible that in the present study, the compression force on the anterior soft tissues was weak between 10° ankle plantar flexion and 0° plantar dorsiflexion and did not differ significantly from that in the healthy group.

Regarding the second finding, in the present study, the shear modulus was compared between the healthy and postoperative groups, and the shear modulus was significantly higher at 3 months and at 6 months in the postoperative group. In addition, Shear modulus decreased in the 6-months compared to the 3 months. Previous studies have reported the presence of extensive fat tissue and synovium anterior to the tibiotalar joint^9^, and these anterior ankle soft tissues have been reported to have a high rate of fibrosis after ankle joint trauma^10,23^, It has also been reported that intra-articular fibrosis was found in 93.9% of patients who presented with gait pain after ankle fracture surgery^24^. A previous study reported that the rate of fibrosis of the IFP increases with increasing stiffness of the IFP^19^. Therefore, the stiffness of anterior ankle soft tissue reflected fibrosis, suggesting that fibrosis may remain at 6 months after ankle fracture surgery.

Regarding the third finding, there were no significant differences in the thickness change ratio and shear modulus between the ankle impingement sign-positive and sign-negative groups. In addition, there was no significant correlation between ankle dorsiflexion ROM and the thickness change ratio or shear modulus. Previous studies on IFP have reported a correlation between anterior knee pain and changes in IFP thickness^21^. It has also been reported that IFP stiffness affects anterior knee pain^19^. This suggests the possibility that both dynamics and stiffness influence IFP pain (anterior knee pain). However, in the present study, the rate of change in anterior ankle soft tissue thickness and shear modulus were not associated with AAIS symptoms and ankle dorsiflexion range of motion. In a previous study, posterior soft tissue contracture was reported as one of the factors contributing to AAIS.B Hickey et al.^25^ reported that posterior ankle soft tissue excision and FHL release by specular surgery improved AAIS symptoms and dorsiflexion range of motion. They suggest that the posterior ankle soft tissue adhesions caused the axis of rotation of the talus to become posterior, contributing to anterior pain. These findings suggest that anterior ankle soft tissue stiffness had little effect on AAIS and dorsiflexion range of motion, and that other factors such as posterior ankle soft tissue may have had an effect.

In the future, it is necessary to approach not only anterior ankle soft tissue stiffness but also other factors such as posterior soft tissue.

This study had three limitations. First, measurement of anterior soft tissue in the final dorsiflexion range of the ankle joint was difficult because of US with probe form issues. Second, the present study did not examine the effect of posterior soft tissue. Third, we did not investigate the period beyond 6 months after surgery. Future studies should examine the effects of posterior soft tissue and long-term follow-up beyond 6 months postoperatively.

## Materials and methods

### Participants

**Figure 1:**
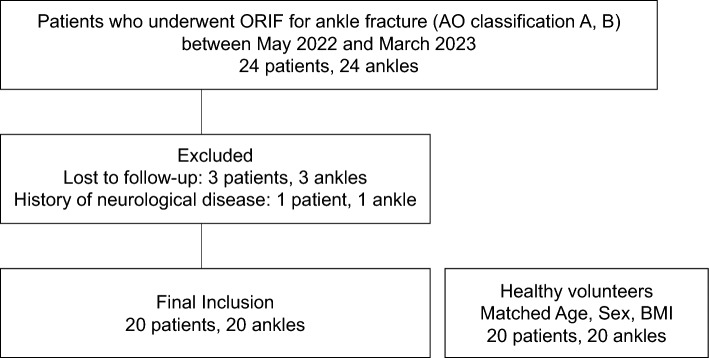
Flow chart detailing the study protocol. *ORIF* open reduction and internal fixation, *BMI* body mass index, *AO* arbeitsgemeinschaft osteosynthesefragen.

Twenty-four patients with ankle fractures (AO classification A, B) who had undergone open reduction and internal fixation (ORIF) between May 2022 and March 2023 were included. Exclusion criteria were: dorsiflexion ROM less than 0°; history of nerve lesions; chronic ankle instability^26^; painful hallux valgus^27^; pronated foot with foot posture index 6 points or higher; and supinated foot with foot posture index 6 points or lower.^28^ Finally, 20 patients (age: 48.5 ± 18.3 years, height: 162.3 ± 8.4 cm, weight: 48.5 ± 18.3 kg) who met the criteria were included as the postoperative group. The healthy group consisted of 20 age and sex-matched subjects (age: 39.2 ± 16.5 years, height: 165.4 ± 7.6 cm, weight: 62.0 ± 11.9 kg). The healthy group was defined as the group without neurological or orthopedic disease (Fig. 1). The postoperative group was evaluated 3 and 6 months postoperatively^17^. After surgery, all patients used crutches for at least the first 2 weeks. After removal of the cast, crutches were used for another 2–4 weeks, although full weightbearing was allowed. All patients underwent a program once a week for three months to train functional skills such as mobility, strength, and stair climbing.

This study was approved by our institution’s ethics review committee (approval number: 3504) and was conducted in accordance with the Declaration of Helsinki. The study was fully explained to the participants in writing and orally, and written, informed consent was obtained from all participants.

### Measurements

**Figure 2:**
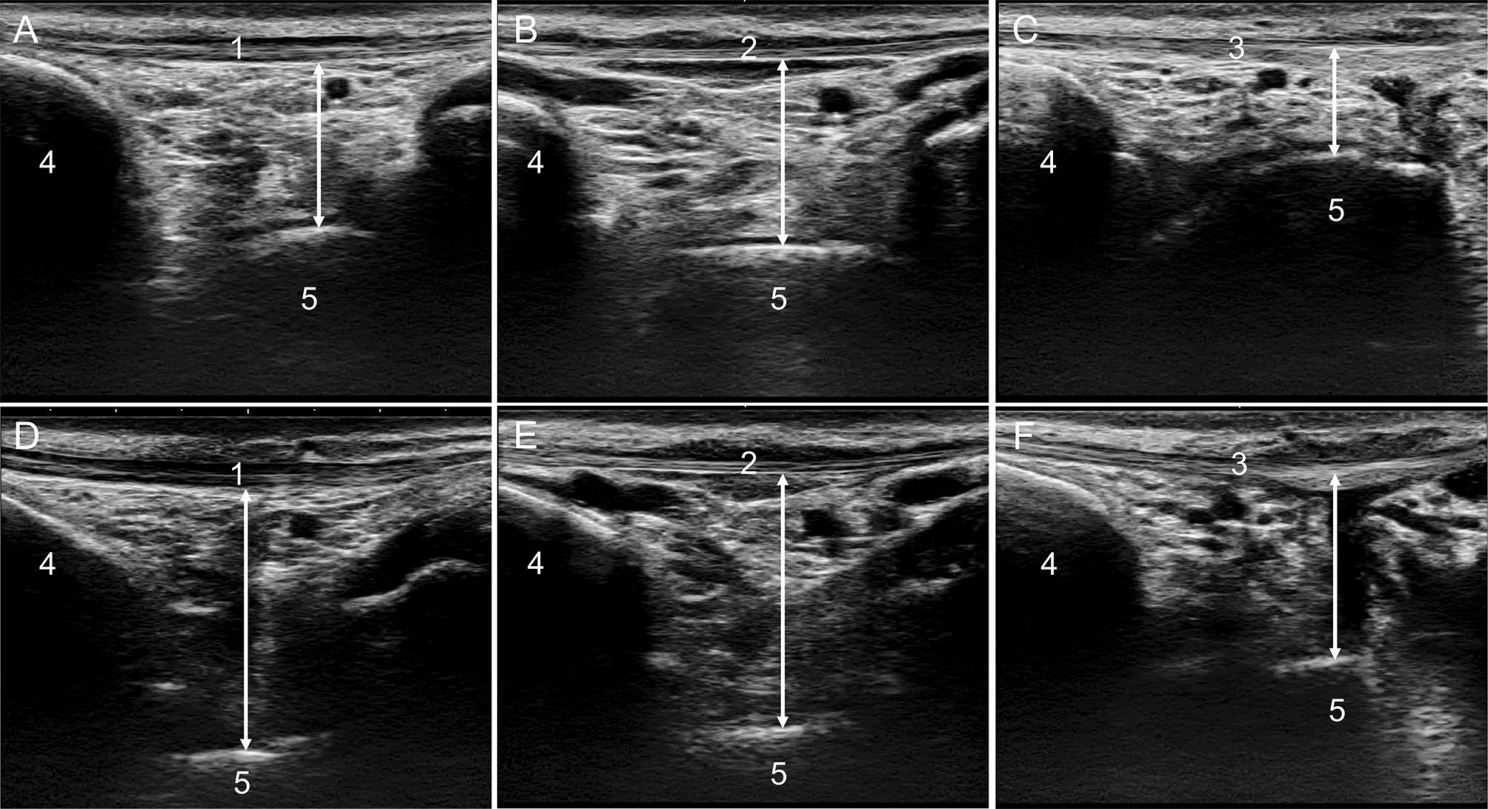
Anterior ankle soft tissue thickness measurement. (**A**) Measurement of the TA tendon at 10° plantarflexion, (**B**) measurement of the EHL tendon at 10° plantarflexion, (**C**) measurement of the EDL tendon at 10° plantarflexion, (**D**) Measurement of the TA tendon at 0° dorsiflexion, (**E**) measurement of the EHL tendon at 0° dorsiflexion, (**F**) measurement of the EDL tendon at 0° dorsiflexion. 1: TA tendon, 2: EHL tendon, 3: EDL tendon, 4: Tibia, (5) Talus.

Anterior ankle soft tissue thickness measurements were performed using a SONIMAGE HS1 (Konica Minolta Corp., Tokyo, Japan) with an 18-MHz high-frequency transducer by a physical therapist with at least 5 years of experience in ultrasound examinations. The position for limb measurements was the resting standing position with the ankle joint in plantarflexion of 10° and dorsiflexion of 0°. The plantarflexion 10° position was defined as 0° knee extension and 10° hip flexion, and the dorsiflexion 0° position was defined as 0° hip extension and 0° knee extension. The participants were set with their feet shoulder-width apart^29^, and their ankle joints were adjusted to avoid excessive pronation and supination^30^. The load was 50% of body weight, the patient was supported by a handrail placed at the level of the greater trochanter to maintain balance, and weight gain or loss was allowed within 5 kg^27,31^. The measurer was careful to apply constant pressure at the lowest level at which ultrasound could be visualized in B mode^32^. For anterior ankle soft tissue measurements, the probe was placed in the long axis over the tibialis anterior (TA), extensor hallucis longus (EHL), and extensor digitorum longus (EDL) tendons, and the talus and tibia were delineated. Anterior ankle soft tissue thickness was defined as the shortest distance from the most convex part of the talus to the tendon directly above it and was measured at each measurement site with the ankle joint in plantarflexion of 10° and dorsiflexion of 0° (Fig. 2). After identifying the measurement site, the movement of each tendon during automatic motion of the ankle joint and toes was confirmed on ultrasound images. The participants were kept stationary, and the images were taken within 30 s at each limb position and measurement site of each tendon. There was a 5-min break in the sitting position at the start of measurement and between measurements on each limb position and each tendon^29^. Measurements of each tendon were performed in random order; each measurement was performed three times and averaged. The anterior ankle soft tissue thickness change ratio was calculated by dividing the value of the limb at 0° plantarflexion by the value at 10° plantarflexion, referring to a previous study by Kitagawa et al.^17^ The intraclass correlation coefficient (ICC) (1,3) was used to evaluate the within-subject reliability of the thickness change ratio measurement for 10 of the subjects (age: 26.2 ± 1.5 years, height: 166.6 ± 7.3 cm, weight: 61.1 ± 14.4 kg). Measurements were taken on different days, spaced about one week apart from the next day. Inter-rater reliability was assessed using the ICC (2,3) for the same subjects as the intra-rater reliability assessment. Inter-rater reliability was assessed by two physical therapists with at least five years of ultrasound experience who took the measurements at random.

**Figure 3:**
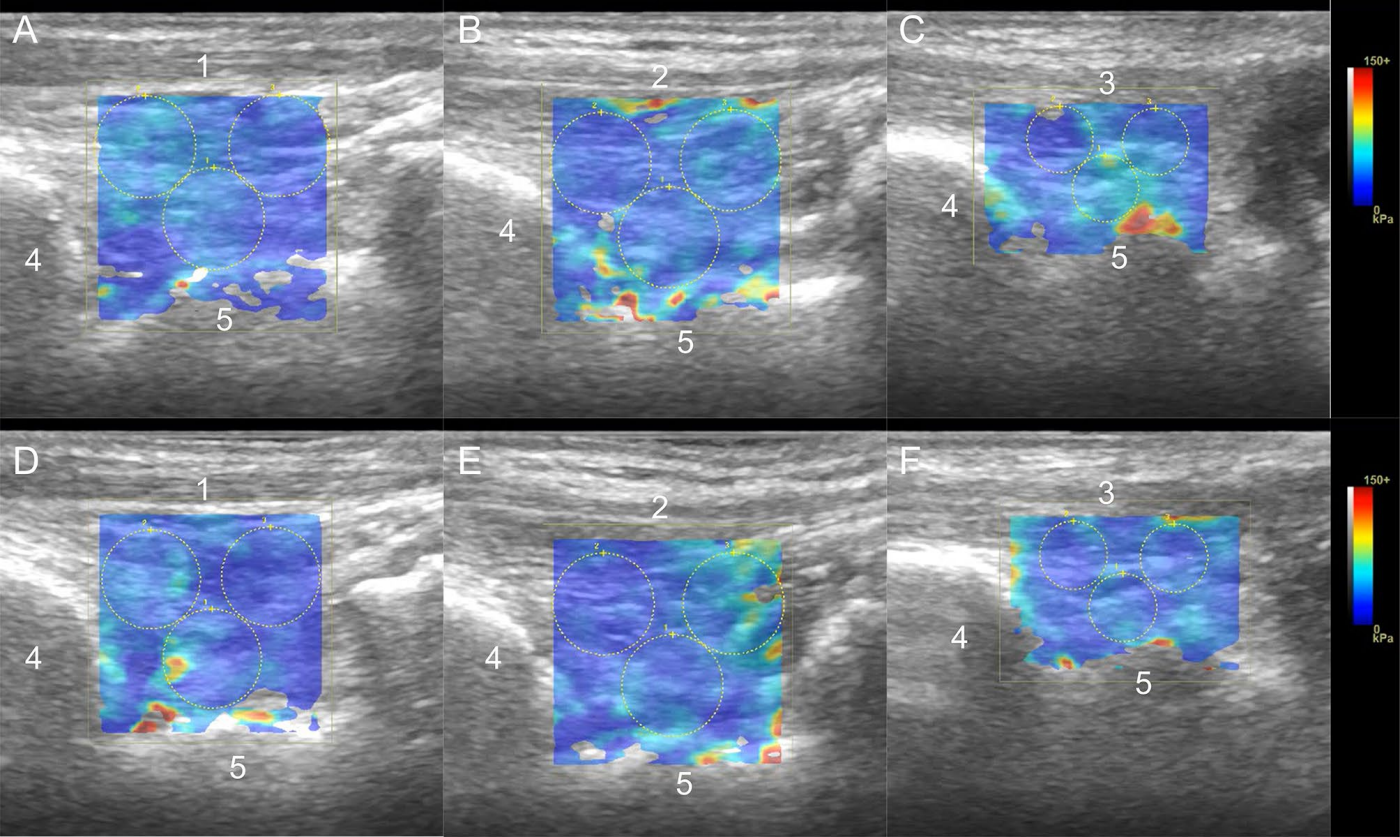
Shear modulus measurement of anterior ankle soft tissue. (**A**) Measurement of the TA tendon at 10° plantarflexion, (**B**) measurement of the EHL tendon at 10° plantarflexion, (**C**) measurement of the EDL tendon at 10° plantarflexion, (**D**) measurement of the TA tendon at 0° dorsiflexion, (**E**) measurement of the EHL tendon at 0° dorsiflexion, (**F**) Measurement of the EDL tendon at 0° dorsiflexion. 1: TA tendon, 2: EHL tendon, 3: EDL tendon, 4: Tibia, 5: Talus.

**Figure 4:**
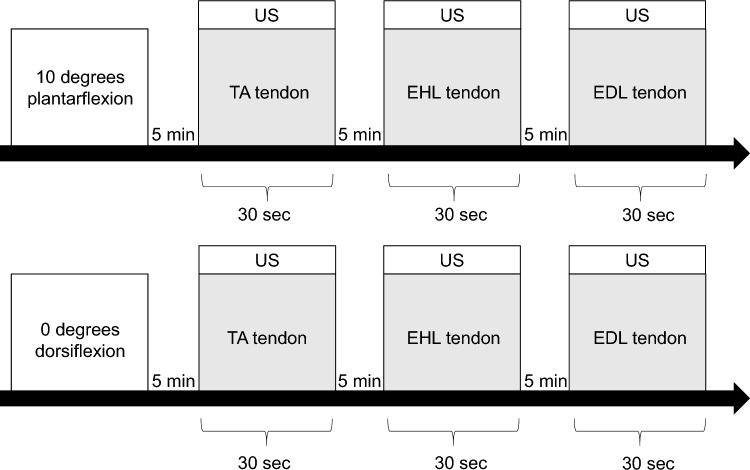
Protocol for measurements on ultrasound. *TA* Tibialis anterior, *EHL* extensor hallucis longus, *EDL* extensor digitorum longus, *US* anterior ankle soft tissue thickness and shear modulus measured using ultrasound imaging system, respectively. Measurements of each limb position and each tendon were taken randomly.

The shear modulus was measured using US (LOGIQ S8, GE Healthcare, Tokyo, Japan) equipped with a linear probe. Shear-wave elastography was used to measure anterior ankle soft tissue stiffness, and long-axis images were drawn similar to thickness measurements. Limb position was measured in the same way as for the anterior ankle soft tissue thickness measurement. Measurements were taken in a random order for each limb and each tendon. The participants were kept stationary, and the images were taken within 60 s at each limb position and measurement site of each tendon. At the beginning of the measurements and between measurements of each limb position and each tendon, a 5-min rest was taken in the sitting position^29^. The largest possible quadrangular region of interest (ROI) was set within the area over the tibia and talus and each tendon^33^. Shear modulus was measured at three points within the ROI, accounting for tendinous artifacts. Shear modulus was measured by placing a circle as large as possible at one deep and two shallow points in the center of the talus^34^. The average shear modulus (kPa) in the three ROIs was calculated (Fig. 3). Shear modulus was measured three times, and the average measured on each tendon was used as the representative value. ICC (1,3) was used to assess intra-rater reliability in 10 subjects with the same anterior ankle soft tissue thickness measurements. For intra-assessor reliability evaluation, the same procedure was performed twice on different days with an interval of about one week from the next day. ICC (2,3) was used to assess inter-rater reliability for the same subjects, as in the intra-rater reliability assessment. In the inter-rater reliability assessment, two physical therapists with at least 5 years of experience in ultrasound performed the measurements randomly (Fig. 4).

The postoperative group was evaluated for the presence of ankle impingement signs, referring to a previous study by Molloy et al.^35^. As in the previous study, a positive ankle impingement sign was defined as tenderness on the anterolateral side of the ankle joint produced or intensified by forced dorsiflexion; those with positive ankle impingement signs constituted the positive group, and those with negative signs the negative group^35^.

The dorsiflexion ROM of the ankle joint was measured using a Todai goniometer (TTM-KO, Sakai Medical, Tokyo, Japan) with the line connecting the head of the fibula and lateral malleolus as the base axis and that between the base and bone head of the fifth metatarsal with the metatarsal head as the axis of motion^36^. The measurer performed passive ROM measurements in a non-loading knee flexion position while keeping the ankle in a neutral position to avoid compensation^36,37^.

### Statistical analysis

Intra-rater and inter-rater reliabilities were calculated with ICC (1,3) and ICC (2,3), interpreted according to the criteria of Landis^38^: < 0.00, poor; 0.00–0.20, slight; 0.21–0.40, fair; 0.41–0.60, moderate; 0.61–0.80, substantial; and 0.81–1.00, almost perfect. The minimal detectable difference at the 95% confidence interval (MDD95%) was calculated as follows: MDD95% = z × SEM × √2, where z = 1.96 and standard error of measurement (SEM) = SD√(1 − ICC)^39^.

The anterior ankle soft tissue thickness change ratio and shear modulus were compared between the healthy and postoperative groups (3 and 6 months) using the no correspondence *t*-test and between the postoperative groups at 3 months and 6 months using the paired *t*-test, with Bonferroni correction as an a posteriori test. In patients with and without AAIS symptoms, the anterior ankle soft tissue thickness change ratio and shear modulus were compared between the positive and negative groups using an unpaired *t*-test. The relationships of ankle dorsiflexion ROM, anterior ankle soft tissue thickness change ratio, and shear modulus in the postoperative ankle fracture group were evaluated using Pearson’s correlation coefficient. All analyses were performed using R (version 2.8.1), with a significance level of 5%.

## Perspective

In this study, the anterior ankle soft tissue thickness change ratio and shear modulus after ankle fracture surgery were investigated. Comparing the healthy and postoperative groups, there was no significant difference in the thickness change ratio, but the shear modulus was significantly higher in the postoperative group. It was suggested that the stiffness of the anterior ankle soft tissue may increase after ankle fracture surgery.

### Ethical approval

The study protocol complied with the principles outlined in the Declaration of Helsinki and was approved by the Ethics Review Board Committee of Keiyu Orthopaedic Hospital (approval number: 3504).

## Data Availability

The data that support the findings of this study are available from the corresponding author upon reasonable request.
